# Duet: SNP-assisted structural variant calling and phasing using Oxford nanopore sequencing

**DOI:** 10.1186/s12859-022-05025-x

**Published:** 2022-11-07

**Authors:** Yekai Zhou, Amy Wing-Sze Leung, Syed Shakeel Ahmed, Tak-Wah Lam, Ruibang Luo

**Affiliations:** Department of Computer Science, The University of Hong Kong, Hong Kong, China

**Keywords:** Structural variant (SV) calling, SV phasing, SV genotyping, SNP calling, Oxford Nanopore Sequencing

## Abstract

**Background:**

Whole genome sequencing using the long-read Oxford Nanopore Technologies (ONT) MinION sequencer provides a cost-effective option for structural variant (SV) detection in clinical applications. Despite the advantage of using long reads, however, accurate SV calling and phasing are still challenging.

**Results:**

We introduce Duet, an SV detection tool optimized for SV calling and phasing using ONT data. The tool uses novel features integrated from both SV signatures and single-nucleotide polymorphism signatures, which can accurately distinguish SV haplotype from a false signal. Duet was benchmarked against state-of-the-art tools on multiple ONT sequencing datasets of sequencing coverage ranging from 8× to 40×. At low sequencing coverage of 8×, Duet performs better than all other tools in SV calling, SV genotyping and SV phasing. When the sequencing coverage is higher (20× to 40×), the F1-score for SV phasing is further improved in comparison to the performance of other tools, while its performance of SV genotyping and SV calling remains higher than other tools.

**Conclusion:**

Duet can perform accurate SV calling, SV genotyping and SV phasing using low-coverage ONT data, making it very useful for low-coverage genomes. It has great performance when scaled to high-coverage genomes, which is adaptable to various clinical applications. Duet is open source and is available at https://github.com/yekaizhou/duet.

## Background

The importance of structural variants (SVs) in genetic disorders has been elaborated in recent studies using traditional molecular techniques [1–3]. Third-generation sequencing (TGS) generates long-reads, which improve the detection resolution down to the base-pair level and are therefore informative enough to achieve phasing [4, 5]. Resolving SV haplotypes can be useful for determining allele-specific expression, compound heterozygosity, and other diplotypic effects [6–10], thus supporting more accurate diagnosis and treatment planning. One of the more cost-effective TGS options for SV detection involves applying the Oxford Nanopore Technologies (ONT) MinION sequencer for whole genome sequencing.

However, current SV detection tools including cuteSV [11], SVIM [12], NanoVar [13], and Sniffles [14] have several constraints. First, the accuracy of SV calling can only be guaranteed when the provided sequencing coverage is high, which is not always available in actual clinical settings. The unsatisfactory performance of existing state-of-the-art tools for low-coverage sequencing data originates from their quality control methods. These tools identify false signals when the number (N_SV_) and the proportion (p_SV_) of supporting reads with SV marks are low [11–14]. This approach works well for high-coverage sequencing data where there are abundant number of supporting reads, and a clear gap in N_SV_ and p_SV_ between the true SV calls and the false signals, which can be observed and used for quality control. However, in low-coverage settings, it is common that there are only one or two supporting reads with SV marks for both true and false signals. Low-coverage data will generate SV candidates with low and very close p_SV_ and N_SV_, which are hard to distinguish from true SVs and false signals based on this single feature. Therefore, novel supporting read signatures and novel features may add to the granularity of the quality control workflow, which is promising for boosting the performance of low-coverage SV calling.

Second, these tools can only distinguish the genotype of the detected SVs (SV genotyping). The genotype can be assigned homozygous (1/1) or heterozygous (0/1) if the level of p_SV_ and N_SV_ is high or median, but the tools lose discriminative power when the sequencing coverage is low. Moreover, this method does not distinguish the paternal (1|0) or maternal (0|1) haplotype of the heterozygous SVs (SV phasing), which is important for clinical applications. To achieve SV phasing and accurate SV genotyping in low-coverage, it is reasonable to solicit additional supporting read signatures, such as the haplotype tendencies of the reads potentially from single-nucleotide polymorphism (SNP) calling and phasing. The additional signatures serve as the raw materials to derive novel features, which provide the granularity for SV phasing and quality control in SV calling at low-coverage.

In this paper, we introduce Duet, a tool for SV calling, genotyping, and phasing, optimized for ONT data. Instead of relying solely on the SV signatures on the reads [11–14], Duet incorporates SNP signatures to observe paternal or maternal tendencies of each SV supporting read. The tool further integrates both SV and SNP signatures into several novel features. The features form as an interpretable and robust decision path, which can characterize SV haplotype from false signals, even when the number of SV supporting reads is moderate. Therefore, while most existing approaches for SV phasing, require both high-coverage [15] and multi-platform sequencing data [16–19], Duet can accurately call and phase SVs with only 8× whole genome sequencing (WGS) ONT data, and has great performance in scaling when the sequencing coverage goes higher, which is promising in various clinical applications.

## Implementation

**Fig. 1:**
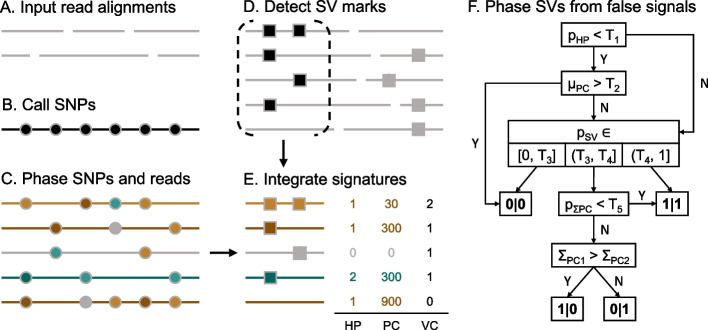
Workflow of Duet. **A** First, ONT long reads are aligned using Minimap2. **B**, **C** To obtain the per-read phasing information (green or brown) with its confidence level (luminance of the color), SNPs (indicated in circles) are called using Clair3 and then phased using WhatsHap. Based on the phased SNPs, the haplotypes of the reads are determined by WhatsHap. **D** The SV marks on each read are detected by cuteSV. **E** Three signatures from previous steps are integrated as the signature of the supporting reads. **F** Duet phases SV and filters out false signals based on the features derived from the signatures in step (**E**). Description of the signatures and features at step (**E**) and step (**F**) is presented in Table 1. T_1_ to T_5_ are thresholds for each feature

**Fig. 2:**
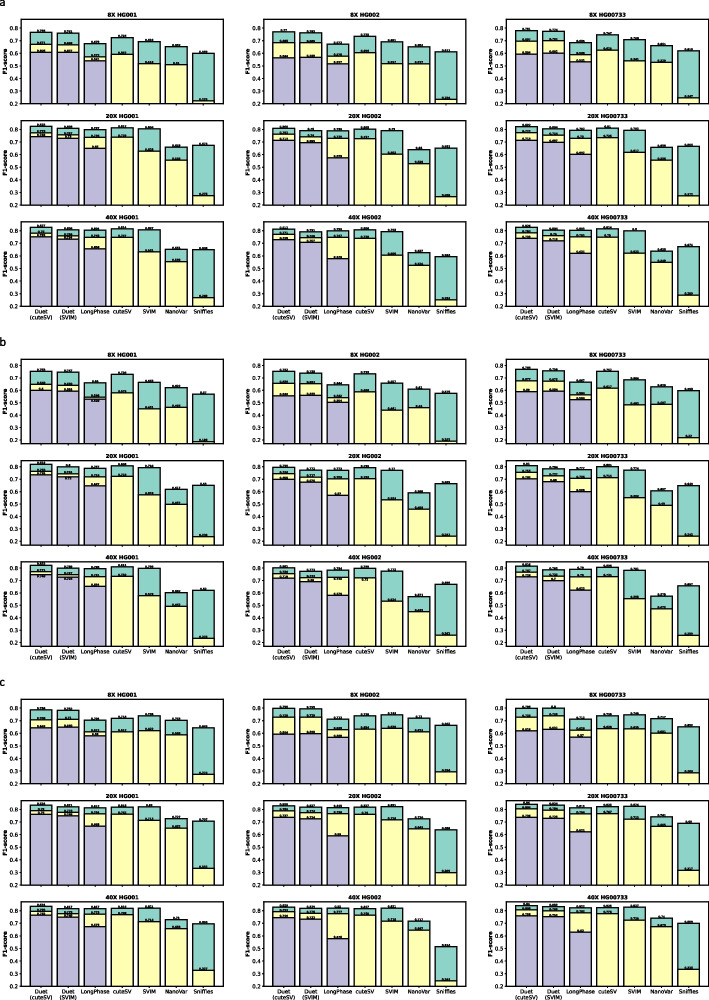
Benchmark results of SV detection tools for ONT data. **a** Benchmark results on all SVs. **b** Benchmark results on insertions. **c** Benchmark results on deletions. Rectangles in green, yellow, and purple colors are performance of SV calling, genotyping, and phasing, respectively

**Fig. 3:**
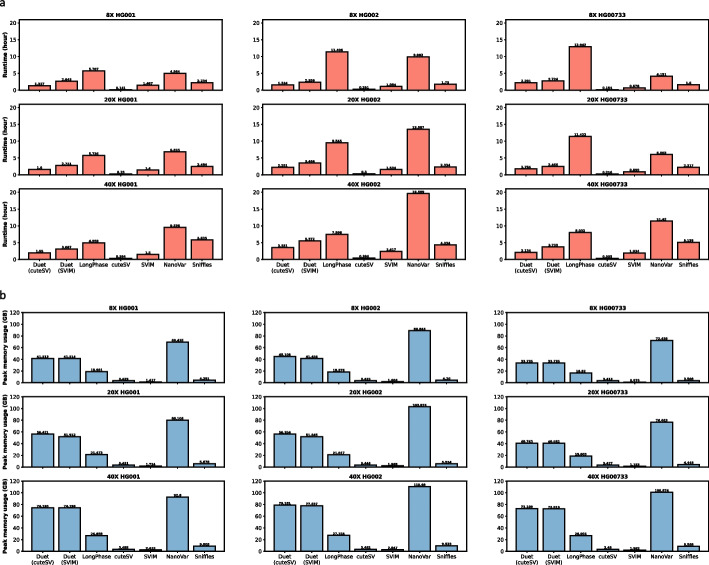
Runtime and peak memory usage of SV detection tools for ONT data with 40 CPU threads. **a** benchmark results on runtime. **b** Benchmark results on peak memory usage

**Fig. 4:**
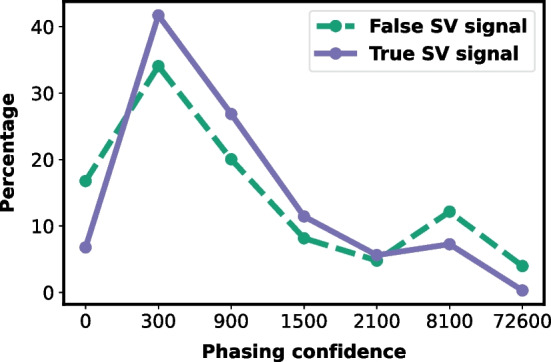
Relationship and distribution of the confidence of the SV signal and the phasing confidence of the supporting read. The x-axis represents phasing confidence in ranges of 0: [0, 300), 300: [300, 900), 900: [900, 1500), 1500: [1500, 8100), 8100: [8100, 72,600), 72,600: [72600, +∞)

The schematic diagram of Duet is depicted in Fig. 1. Taking a long-read alignment [20] and its reference as input (Fig. 1A), Duet processes the data with four major modules, comprising (1) SNP calling (Fig. 1B), (2) SNP phasing and per-read haplotype assignment (Fig. 1C), (3) SV calling (Fig. 1D), and (4) SV phasing (Fig. 1E, F) to output phased SVs. The tool is designed to tolerate false positives of low-coverage ONT WGS data while retaining a high level of accuracy, although the use of a high-coverage dataset (i.e., ≥ 20×) can enhance its performance.

**Table 1:** Signatures and features used by Duet

Supporting read signature	
HP	Haplotype
PC	Confidence score of the haplotype prediction
VC	Count of SV marks

Feature for SV phasing	
p_SV_ = Σ_VC>0_/Σ_VC ≥ 0_	Proportion of reads with SV marks
p_HP_ = Σ_VC,HP>0_/Σ_VC>0_	Proportion of phasable reads with SV marks
Σ_PC_ = Σ_VC*PC per HP_	Total PC for read set from each haplotype
μ_PC_ = μ_VC*PC per HP_	Average PC for read set from each haplotype
p_ΣPC_ = Σ_PC1_/Σ_PC2_	Ratio of Σ_PC_ for both haplotype

### SNP calling

SNP calling generates heterozygous SNPs that can be used as a proxy to infer the reads’ haplotypes. We applied the ONT model of Clair3 [21], a fast and accurate long-read small-variant caller, with “--snp_min_af 0.25 --pileup_only --call_snp_only” options after experimenting with different settings. The settings were fine-tuned in Duet, which increased the overall processing speed, in addition to providing sufficient variant-calling accuracy for phasing.

### SNP phasing and per-read haplotype assignment

Using the SNP-calling results, Duet applies WhatsHap [22] with “whatshap phase --distrust-genotypes” to generate the two parental haplotypes. The settings provide WhatsHap with higher tolerance for false positives, especially when working with low-coverage datasets. The haplotag “whatshap haplotag --tag-supplementary” subfunction is then called to assign haplotype to each read. In addition, each read is assigned a confidence score by WhatsHap, which is positively related to the number of accurately phased variants. Duet will use the haplotype and the prediction confidence of the reads. We employ GNU Parallel [23] to allow parallel processing of all chromosomes.

### SV calling

It is essential to apply a base SV caller that is sensitive even when using low-coverage data to reduce the initial false-negative rate, as a large proportion of false positives can be filtered out after SV phasing. After performance evaluation, cuteSV [11] with the setting “-s 2 --max_cluster_bias_INS 100 --diff_ratio_merging_INS 0.3 --max_cluster_bias_DEL 200 --diff_ratio_merging_DEL 0.5” provides the best output to fit the purpose of Duet, where the SV marks detected are loosely clustered for downstream analysis.

### SV phasing

Duet integrates the SV and SNP signatures obtained from the above modules to represent each supporting read, i.e., haplotype (HP), confidence of the haplotype prediction (phasing confidence, PC), and the number of SV marks (VC). For each SV call set with multiple supporting reads, several novel features are derived from the signatures, and an empirical rule-based decision path with corresponding cut-off values is derived accordingly.

The decision path contains three layers:

First, an initial filter is applied to filter out SV calls that contain too small a proportion of phasable reads (p_HP_) with a very high average phasing confidence (μ_PC_).

The remaining SV calls will go through a subsequent characterization based on the proportion of reads containing SV marks (p_SV_), to identify false positive SV calls (0/0) if with too low p_SV_, and homozygous variants (1|1) if with high p_SV_.

The further remaining SV calls with moderate proportion of reads with SV marks will be finally distinguished between being homozygous or heterozygous, where the phasing confidence is taken into meticulous consideration: Duet classifies the supporting reads into paternal group and maternal group based on their haplotype. Then the sum of the phasing confidence for each haplotype group is calculated (Σ_PC1_, Σ_PC2_). If these two values have no huge difference (i.e., a moderate p_ΣPC_), the SV call will be classified as homozygous (1|1). Otherwise, if there is a huge difference between the total phasing confidence, the SV call will be classified as heterozygous and assigned to the haplotype with the dominant total phasing confidence (0|1 or 1|0).

### Evaluation methods and metrics

We have an integrated evaluation method for SV calling, genotyping, and phasing, with code available at [https://github.com/yekaizhou/duet/blob/main/src/scripts/evaluation.py]. The evaluation criteria of SV calling and genotyping is adapted from Truvari [https://github.com/ACEnglish/truvari].

In the evaluation of SV calling, an SV candidate is determined as a true positive (TP) if it meets the following conditions:

1 $$\left| {{\text{comp}}_{{\text{s}}} - {\text{base}}_{{\text{s}}} } \right| \le {1}\;{\text{kbp}}$$

$${\text{min}}({\text{comp}}_{{\text{L}}} ,{\text{base}}_{{\text{L}}} ){\text{/max}}({\text{comp}}_{{\text{L}}} ,{\text{base}}_{{\text{L}}} ) \ge 0.{4}$$

$${\text{comp}}_{{\text{t}}} = {\text{base}}_{{\text{t}}}$$

where comp_s_, comp_L_ and comp_t_ are the start coordinate, length, and the class of an SV prediction, respectively, while the base_s_, base_L_ and base_t_ are the starting coordinate, length, and the class of an SV recorded in the truth set, respectively. An SV call is counted as a false positive (FP) if it does not satisfy Eq. 1. A ground truth SV is assigned as a false negative (FN) if there is no SV call that satisfies Eq. (1) with it.

With the above definition, precision (or the ratio of TPs to total calls in predictions) is defined as:

2 $${\text{Precision}} = {\text{TPs/}}({\text{TPs}} + {\text{FPs}})$$

Recall (or the ratio of TPs to total calls in the truth set) is defined as:

3 $${\text{Recall}} = {\text{TPs/}}({\text{TPs}} + {\text{FNs}})$$

F1 score is defined as:

4 $${\text{F1}} = {2} \times {\text{Precision}} \times {\text{Recall/}}({\text{Precision}} + {\text{Recall}})$$

When considering the performance of SV genotyping, each TP SV call in SV calling gets further evaluated if its genotype is the same as the corresponding base call. If so, it is assigned to be a TP call, otherwise, it will be assigned as an FP call. All the FP calls in the SV calling evaluation procedure remain FP in SV genotyping. Each of the ground truth SVs will be evaluated and assigned TP if there is at least one TP call corresponding to it, otherwise it is assigned false negative (FN). Equations (2)–(4) are used to calculate the statistics of SV genotyping.

Switch error rate is a standard metric to evaluate the phasing accuracy of abundant and adjacent genome variants such as SNPs. However, this metric fails to be a reasonable metric for SVs, which are large and distant [24]. Therefore, we adapted and adjusted the standard evaluation method of SV genotyping for SV phasing. SV phasing will separately evaluate every phase block produced by WhatsHap: For each phase set, the haplotype prediction of TP SV calls in SV calling will have two versions, one remains original and the other was flipped (0|1 to 1|0; 1|0 to 0|1; 0|0 and 1|1 remains unchanged), and their haplotypes will be separately compared to the ground truth set: If the haplotype matches, it will be a TP call, otherwise, it will be an FP call. All the FP calls in the SV calling evaluation procedure remain FP in SV phasing. The evaluation will record the version with more TPs for every phase set. Each of the ground truth SVs will be evaluated and assigned TP if there is at least one TP call corresponding to it, otherwise it is assigned false negative (FN). Equations (2)–(4) were used to calculate the statistics of SV phasing.

## Results

We compared Duet against four state-of-the-art SV callers, SVIM [12], cuteSV [11], NanoVar [13], and Sniffles [14], for SV calling and genotyping. We also compared Duet against LongPhase [24], an algorithm that can phase SVs based on an existing SV call set generated from other programs. We use the SV call set from cuteSV, and the SNP call set from Clair3 and WhatsHap as the input for LongPhase. In addition to the default setting of using cuteSV as the base SV caller of Duet, we evaluated the performance of Duet using SVIM as the base SV caller. The benchmarking was on HG001, HG002, and HG00733, three standard human samples from the Human PanGenomics Project [25], with available high-confidence haplotype-resolved SV truth sets from HGSVC2 [18]. We tested the performance on three sequencing coverages: 8× (low-coverage), 20× (middle-coverage), and 40× (high-coverage).

The F1-scores are shown in Fig. 2a. At 8× sequencing coverage of the three human samples, Duet achieved up to 0.85 precision, 0.72 sensitivity, and 0.78 F1-score genome-wide for SV calling; 0.74 precision, 0.66 sensitivity, and 0.70 F1-score for SV genotyping; and 0.65 precision, 0.57 sensitivity, and 0.61 F1-score for SV phasing. Duet outperformed other tools in SV calling, genotyping, and phasing at the low coverage of 8×.

The performance of Duet has stable improvement when the sequencing coverage is increased to 20×, and achieved up to 0.87 precision, 0.79 sensitivity, and 0.82 F1-score genome-wide for SV calling; 0.81 precision, 0.75 sensitivity, and 0.77 F1-score for SV genotyping; and 0.77 precision, 0.72 sensitivity, and 0.74 F1-score for SV phasing. Duet showed increased improvement compared to other tools in SV phasing. Duet still outperformed other tools in SV calling and genotyping and the second in place is cuteSV and LongPhase, respectively.

The performance of Duet has slight improvement when the sequencing coverage is increased from 20× to 40×, and achieved up to 0.87 precision, 0.79 sensitivity, and 0.83 F1-score genome-wide for SV calling; 0.82 precision, 0.75 sensitivity, and 0.78 F1-score for SV genotyping; and 0.78 precision, 0.73 sensitivity, and 0.75 F1-score for SV phasing. Duet still has a great competing edge in SV phasing. Duet still outperformed other tools in SV calling and genotyping and the second in place is cuteSV and LongPhase, respectively.

We further benchmarked Duet against the other tools at all three sequencing coverages on specific SV types including insertion and deletion. The results are shown in Fig. 2b, c. Similar to our previous observations on all SVs, Duet outperformed the other tools on both insertion and deletion. Compared to using cuteSV as Duet’s base SV caller, the performance of using SVIM is comparatively lower on our benchmarking samples, albeit Duet with either caller outperformed the other tools. As it is possible for Duet with SVIM to outperform Duet with cuteSV on different samples, we allow users to choose to use either caller in the Duet, with cuteSV as the default option.

The runtime of Duet using 40 CPU threads ranged from 1.3 to 2.2 h at 8×, 1.6 to 2.3 h at 20×, and 2.0 to 3.6 h at 40× sequencing coverage across the three samples (Fig. 3a). Duet uses either cuteSV or SVIM as its base SV caller, therefore it runs longer than both callers. However, Duet still ran faster than LongPhase, NanoVar, and Sniffles. While the average memory usage was lower, the peak memory of Duet ranged from 34 to 45 GB at 8×, 41 to 56 GB at 20×, and 73 to 79 GB at 40× (Fig. 3b).

To demonstrate why SNP signatures are informative for SV detection, we compared the ratio of false and true SV signals with different phasing confidence of the supporting reads. We ran SVIM, Clair3, and WhatsHap on 8× of HG001, HG002, and HG00733 to gather the raw SV call set, haplotype, and phasing confidence of each sequencing read. Each SV candidate was tagged either false positive or true positive using the evaluation method above. Each supporting read of each SV candidate was tagged with a phasing confidence. All the supporting reads of all SV candidates from the three samples were pooled together to derive the relationship between SV signal correctness and the phasing confidence of the supporting read. The results are shown in Fig. 4. We found that a true SV signal happened more often when supporting reads are with a moderate phasing confidence (300 to 2100), but less happened when with a low phasing confidence (0 to 300). Conversely, excessively high phasing confidence (8100 or above) is associated with more false SV signals. The above complications comprise Duet’s decision-making procedure to distrust SV calls with supporting reads having either too low or too high phasing confidence.

**Fig. 5:**
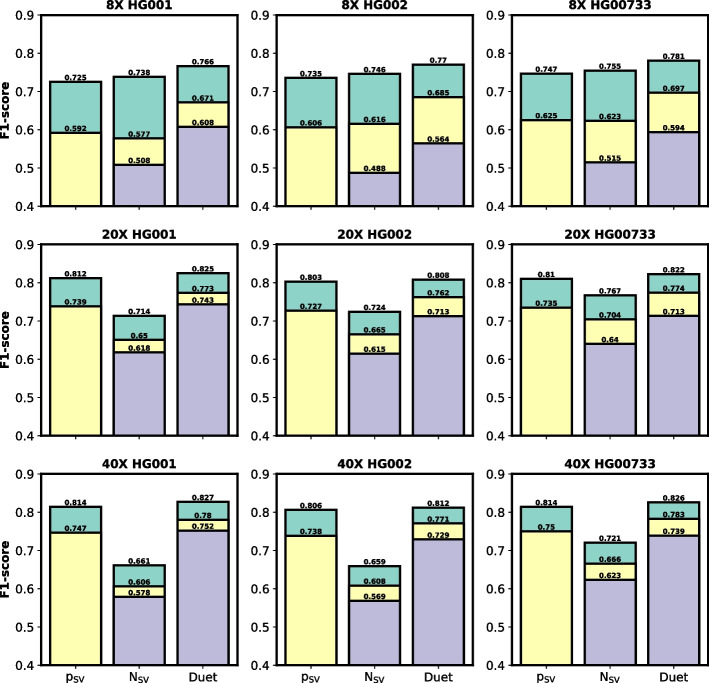
Benchmark results of Duet against two approaches using p_SV_ and N_SV_. p_SV_ is the approach that uses p_SV_ as the threshold to determine SV genotype. N_SV_ is the approach that uses N_SV_ as the threshold to determine SV haplotype. Rectangles in green, yellow, and purple colors are performance of SV calling, genotyping, and phasing, respectively

To demonstrate the advantage of Duet’s novel features compared to the traditionally used features, we additionally compared Duet to using either p_SV_ or N_SV_ for removing false signals. For SV genotyping, we set SV candidates with p_SV_ < 0.2 as false, p_SV_ > 0.8 as homozygous (1/1), and in between as heterozygous (0/1). For SV phasing, we set N_SV_ ≥ 2 for both haplotypes as homozygous (1|1), N_SV_ ≥ 2 for only one haplotype as heterozygous (0|1 or 1|0), and N_SV_ < 2 for both haplotypes as false. The results are shown in Fig. 5. Duet outperformed using the traditional features for false signal detection with all three coverages and three samples.

## Discussion

In this study we developed a tool for SV calling, genotyping, and phasing, which we named Duet. We demonstrated that Duet is more accurate and powerful than other state-of-the-art tools in resolving genomic SVs, particularly those that lead to disease, when only low-coverage data is available, which is very useful in clinical settings and resource-constrained lab settings where sufficient genomic data coverage may not be available. At higher coverages, Duet’s performance in SV phasing is further improved in comparison to other competing tools used for the purpose, while maintaining a greater performance in SV genotyping and SV calling. The benchmark results of Duet further demonstrate that by incorporating SNP calling, SNP phasing, read haplotagging, the tailored decision tree is beneficial to SV detection, especially in accurate SV phasing and the quality control for low-coverage SV calling. Although Duet was optimized for ONT data, it has no restriction applying to PacBio data, albeit some thresholds need to be tuned to achieve the best potential.

There are still some limitations in Duet:

1. Starting from middle-coverage, the competing edge of Duet in SV calling becomes less significant. The main reason is that when the sequencing coverage goes higher, the feature p_SV_ used by other tools has recovered its discriminative power to remove false signals. Duet’s current quality control method has clear improvement at low-coverage, and will generally be caught up by other state-of-the-art methods when the coverage goes higher.
2. The performance improvement of Duet from middle-coverage to high-coverage is moderate. All the benchmarked tools have similar situation. This is because our highly accurate truth sets are derived from multi-platform sequencing, and some genome regions are hard to map using single-strand sequencing technology, even with high coverage. Despite this objective explanation, there is possibly room to improve the performance of Duet at high-coverage data by updating the quality control method to fit both low- and high-coverage sequencing data in a coverage-dependent manner.

## Conclusions

In this study, we introduced Duet, a long-read-based tool for accurate SV calling and phasing. Duet showed promising results that the incorporation of SNP signatures will largely boost the performance of SV detection, especially when there is constraint in sequencing coverage and when there is a need for accurate SV phasing. The tool’s great adaptability and scaling performance in SV calling and phasing promise its usefulness in routine clinical applications.

## Data Availability

The source code of Duet and the scripts for data preparation and benchmarking are available at https://github.com/yekaizhou/duet. The hg38_NoALT human reference genome is available at: http://ftp.1000genomes.ebi.ac.uk/vol1/ftp/data_collections/HGSVC2/technical/reference/20200513_hg38_NoALT/hg38.no_alt.fa.gz. The HG001, HG002, and HG00733 sequencing reads datasets can be downloaded from: https://s3-us-west-2.amazonaws.com/human-pangenomics/NHGRI_UCSC_panel/HG001/nanopore/Guppy_4.2.2/, https://s3-us-west-2.amazonaws.com/human-pangenomics/NHGRI_UCSC_panel/HG002/nanopore/Guppy_4.2.2/, https://s3-us-west-2.amazonaws.com/human-pangenomics/NHGRI_UCSC_panel/HG00733/nanopore/Guppy_4.2.2/. The corresponding ground truth set for these three samples can be downloaded from: http://ftp.1000genomes.ebi.ac.uk/vol1/ftp/data_collections/HGSVC2/release/v2.0/integrated_callset/variants_freeze4_sv_insdel_sym.vcf.gz.

## Abbreviations

FP: False positive
MAF: Minor allele frequency
ONT: Oxford Nanopore Technologies
SNP: Single-nucleotide polymorphism
SV: Structural variant
TP: True positive
WGS: Whole genome sequencing

## Availability and requirements

Project name: Duet

Project home page: https://github.com/yekaizhou/duet

Operating system(s): Platform independent

Programming language: Python

Other requirements: All software requirements are listed in https://github.com/yekaizhou/duet#dependencies

License: BSD-3-Clause

Any restrictions to use by non-academics: None

## References

[1] Sebat J, Lakshmi B, Malhotra D, Troge J, Lese-Martin C, Walsh T, Yamrom B, Yoon S, Krasnitz A, Kendall J (2007). Strong association of de novo copy number mutations with autism. Science.

[2] Stankiewicz P, Lupski JR (2010). Structural variation in the human genome and its role in disease. Annu Rev Med.

[3] Weischenfeldt J, Symmons O, Spitz F, Korbel JO (2013). Phenotypic impact of genomic structural variation: insights from and for human disease. Nat Rev Genet.

[4] Edge P, Bafna V, Bansal V (2017). HapCUT2: robust and accurate haplotype assembly for diverse sequencing technologies. Genome Res.

[5] Patterson M, Marschall T, Pisanti N, van Iersel L, Stougie L, Klau GW, Schonhuth A (2015). WhatsHap: weighted haplotype assembly for future-generation sequencing reads. J Comput Biol.

[6] Browning SR, Browning BL (2011). Haplotype phasing: existing methods and new developments. Nat Rev Genet.

[7] Ge B, Pokholok DK, Kwan T, Grundberg E, Morcos L, Verlaan DJ, Le J, Koka V, Lam KC, Gagne V (2009). Global patterns of cis variation in human cells revealed by high-density allelic expression analysis. Nat Genet.

[8] Glusman G, Cox HC, Roach JC (2014). Whole-genome haplotyping approaches and genomic medicine. Genome Med.

[9] Roach JC, Glusman G, Smit AF, Huff CD, Hubley R, Shannon PT, Rowen L, Pant KP, Goodman N, Bamshad M (2010). Analysis of genetic inheritance in a family quartet by whole-genome sequencing. Science.

[10] Tewhey R, Bansal V, Torkamani A, Topol EJ, Schork NJ (2011). The importance of phase information for human genomics. Nat Rev Genet.

[11] Jiang T, Liu Y, Jiang Y, Li J, Gao Y, Cui Z, Liu Y, Liu B, Wang Y (2020). Long-read-based human genomic structural variation detection with cuteSV. Genome Biol.

[12] Heller D, Vingron M (2019). SVIM: structural variant identification using mapped long reads. Bioinformatics.

[13] Tham CY, Tirado-Magallanes R, Goh Y, Fullwood MJ, Koh BTH, Wang W, Ng CH, Chng WJ, Thiery A, Tenen DG (2020). NanoVar: accurate characterization of patients' genomic structural variants using low-depth nanopore sequencing. Genome Biol.

[14] Sedlazeck FJ, Rescheneder P, Smolka M, Fang H, Nattestad M, von Haeseler A, Schatz MC (2018). Accurate detection of complex structural variations using single-molecule sequencing. Nat Methods.

[15] Chin CS, Peluso P, Sedlazeck FJ, Nattestad M, Concepcion GT, Clum A, Dunn C, O'Malley R, Figueroa-Balderas R, Morales-Cruz A (2016). Phased diploid genome assembly with single-molecule real-time sequencing. Nat Methods.

[16] Chaisson MJP, Sanders AD, Zhao X, Malhotra A, Porubsky D, Rausch T, Gardner EJ, Rodriguez OL, Guo L, Collins RL (2019). Multi-platform discovery of haplotype-resolved structural variation in human genomes. Nat Commun.

[17] Cretu Stancu M, van Roosmalen MJ, Renkens I, Nieboer MM, Middelkamp S, de Ligt J, Pregno G, Giachino D, Mandrile G, Espejo Valle-Inclan J (2017). Mapping and phasing of structural variation in patient genomes using nanopore sequencing. Nat Commun.

[18] Ebert P, Audano PA, Zhu Q, Rodriguez-Martin B, Porubsky D, Bonder MJ, Sulovari A, Ebler J, Zhou W, Serra Mari R (2021). Haplotype-resolved diverse human genomes and integrated analysis of structural variation. Science.

[19] Rodriguez OL, Ritz A, Sharp AJ, Bashir A (2020). MsPAC: a tool for haplotype-phased structural variant detection. Bioinformatics.

[20] Li H (2018). Minimap2: pairwise alignment for nucleotide sequences. Bioinformatics.

[21] Zheng Z, Li S, Su J, Leung AW-S, Lam T-W, Luo R. Symphonizing pileup and full-alignment for deep learning-based long-read variant calling. *bioRxiv* 2021:2021.2012.2029.474431. 10.1038/s43588-022-00387-x 38177392

[22] Martin M, Patterson M, Garg S, O Fischer S, Pisanti N, Klau GW, Schöenhuth A, Marschall T. WhatsHap: fast and accurate read-based phasing. *bioRxiv* 2016:085050.

[23] Tange O (2011). GNU Parallel-The Command-Line Power Tool. USENIX Mag.

[24] Lin JH, Chen LC, Yu SC, Huang YT (2022). LongPhase: an ultra-fast chromosome-scale phasing algorithm for small and large variants. Bioinformatics.

[25] Shafin K, Pesout T, Lorig-Roach R, Haukness M, Olsen HE, Bosworth C, Armstrong J, Tigyi K, Maurer N, Koren S (2020). Nanopore sequencing and the Shasta toolkit enable efficient de novo assembly of eleven human genomes. Nat Biotechnol.

